# Decrease in the Crystallite Diameter of Solid Crystalline Magnetite around the Curie Temperature by Microwave Magnetic Fields Irradiation

**DOI:** 10.3390/nano11040984

**Published:** 2021-04-11

**Authors:** Takayuki Tsuchida, Jun Fukushima, Hirotsugu Takizawa

**Affiliations:** School of Engineering, Department of Applied Chemistry, Tohoku University, Sendai 980-8578, Japan; fukushima@aim.che.tohoku.ac.jp (J.F.); takizawa@aim.che.tohoku.ac.jp (H.T.)

**Keywords:** de-crystallization, microwave processing, magnetite, Curie temperature, non-thermal effects, ferrites

## Abstract

A decrease in the crystallite diameter of ferrites irradiated with microwaves has been considered as a non-thermal effect of so-called de-crystallization; however, its mechanism has not been elucidated. We hypothesized that a decrease in the crystallite diameter is caused by interaction between the ordered spins of ferrite and the magnetic field of microwaves. To verify this, we focused on magnetite with a Curie temperature of 585 °C. Temperature dependence around this temperature and time dependence of the crystallite diameter of the magnetite irradiated with microwaves at different temperatures and durations were investigated. From the X-ray diffraction data, the crystallite diameter of magnetite exhibited a minimum value at 500 °C, just below the Curie temperature of magnetite, where the energy loss of the interaction between magnetite’s spins and the microwaves takes the maximum value. The crystallite diameter exhibited a minimum value at 5 min irradiation time, during which the microwaves were excessively absorbed. Transmission electron microscopy observations showed that the microstructure of irradiated magnetite in this study was different from that reported previously, indicating that a decrease in the crystallite diameter is not caused by de-crystallization but its similar phenomenon. A decrease in coercivity and lowering temperature of Verwey transition were observed, evidencing decreased crystallite diameter. This study can thus contribute to the development of the theory of a non-thermal effect.

## 1. Introduction

Microwave processing exhibits some unique features, such as rapid heating, rapid cooling, and selective heating, which are different from conventional processing using an electric furnace. Thus, microwave heating provides many benefits for material processing, such as decreasing the sintering time, synthesizing functionalized nanomaterials, and promoting catalytic reactions [1,2,3,4,5,6,7,8,9]. Additionally, “non-thermal effects”, phenomena of which cannot be explained by thermal effects, have also been reported [10,11,12,13,14,15,16,17]. In the case of solid-state reactions, reported non-thermal effects include promoting the reduction of oxides [10,11], nitriding [12], as well as anisotropic growth [13]. Although these studies indicate unique phenomena due to microwave processing, non-thermal effects are controversial because the mechanism of many of them has still not been clarified.

De-crystallization is one of the phenomena considered to be a non-thermal effect [18,19,20,21,22,23,24,25,26]. This phenomenon was first discovered by Kimura et al. in 2000; X-ray diffraction showed halo patterns for ferrites irradiated with 28 GHz frequency [18]. In addition to ferrites, de-crystallization of n-type Si [22,23], TiO_2−*x*_, and Ti_0.98_Mn_0.02_O_2−x_ [24] have been reported. Takayama et al. used transmission electron microscopy to investigate the microstructure of magnetite (Fe_3_O_4_) irradiated with microwaves, and a 5–20 nm nanodomain structure was observed [25]. In particular, de-crystallization of the ferrites by microwave irradiation is considered as a non-thermal effect for the following two reasons [21]: First, de-crystallization does not occur via the liquid phase. Second, de-crystallization irradiated with an electric field of microwaves in the single-mode cavity has not been confirmed despite the same temperature processing. These results suggest that de-crystallization is a non-thermal effect; however, the mechanism of de-crystallization has not been determined. We hypothesized that de-crystallization of the ferrites is caused by an interaction between the ordered spins of ferrites and a microwave magnetic field because this phenomenon only occurs under the magnetic field of the single-mode cavity. To verify this hypothesis, the authors focused on magnetite, with a Curie temperature, *T*_C_, of 585 °C as the ferrite and investigated the temperature dependence of the interaction between the ordered spins of magnetite and a microwave magnetic field. As a result, a decrease in the crystallite diameter of magnetite was observed, which is not considered to be de-crystallization but its similar phenomenon. In addition, the effect of the duration of microwave irradiation on the crystallite diameters of magnetite was investigated to understand the dynamics of this phenomenon.

## 2. Materials and Methods

**Figure 1:**
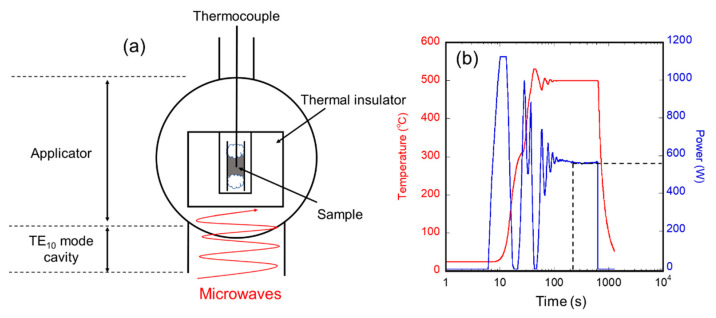
(**a**) Schematic of the experimental setup; (**b**) Temperature and microwave power profile during processing.

Figure 1a shows a schematic view of the experimental setup used in this study. We used a 2.45 GHz microwave furnace (Shikoku Instrumentation Co., Ltd., Kagawa, Japan), as used in [16] and [27], in which a microwave was generated from a magnetron and passed through a TE_10_ waveguide to a cylindrical applicator (Ø 132 × 200 mm). In the applicator, the *H*-field and *E*-field components of microwaves have different distributions, and we used the 3 cm height position where the *H*-field amplitude was at its maximum. For the raw materials, we selected magnetite powders that exhibit de-crystallization phenomena [21]. Magnetite powder (0.3 g) (99% purity, 1 mm pass, Kojundo Chemical Lab. Co., Saitama, Japan) was placed in a Ø 10 × 8 mm quartz tube and sealed with quartz wool. The tube was surrounded by an alumina insulator. The microwave cavity was evacuated using a rotary pump and subsequently filled with nitrogen gas. A Proportional-Integral-Differential (PID) controller was used to automatically adjust the microwave power to the desired temperature, which was measured using a thermocouple. Each sample was heated from room temperature to a certain constant temperature, which was chosen in the range of 200–1100 °C, for a certain duration, which was chosen in the range of 0.2–60 min, after heating at 900 °C/min. After the holding time, the samples were naturally cooled by deactivating the microwave power. As an example, the temperature and power profiles of heating at 500 °C for 60 min are shown in Figure 1b. The samples irradiated with microwaves were characterized by X-ray diffraction (XRD) with a Co Kα source (*λ* = 1.78900 Å), and the crystallite diameters, which is the size of single-crystal regions, of the samples were calculated using the Scherrer equation. The microstructure of the samples irradiated with microwaves was observed by transmission electron microscopy (TEM), and the magnetic properties of the resulting samples were evaluated by superconducting quantum interference device (SQUID) measurements.

## 3. Results

### 3.1. Structure

**Figure 2:**
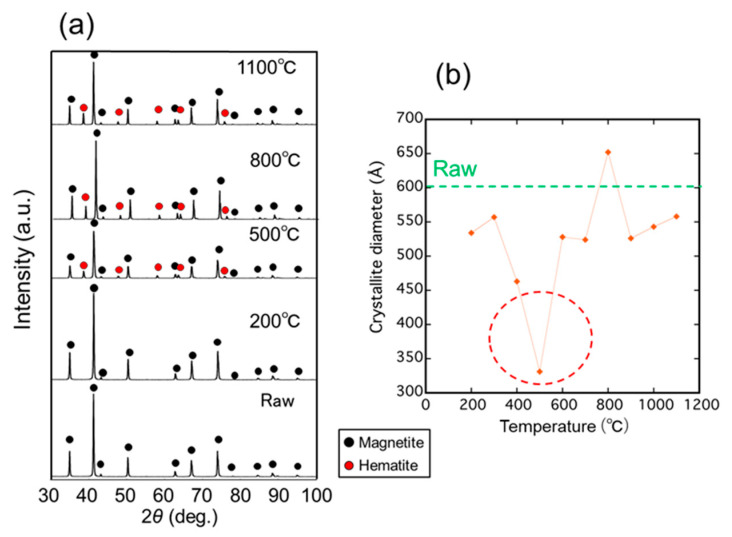
(**a**) XRD patterns of the samples irradiated with microwave at different temperatures for 5 min; (**b**) Crystallite diameter of the magnetite calculated by the Scherrer equation as a function of irradiation temperature.

Figure 2a shows the XRD profiles of the samples irradiated with microwaves at various temperatures from 200 to 1100 °C for 5 min. Diffraction peaks due to magnetite were clearly observed in the samples irradiated with microwaves and the raw material. However, diffraction peaks due to hematite (*α*-Fe_2_O_3_) were only observed in samples irradiated with microwaves above 300 °C, indicating partly oxidized samples. Figure 2b shows the crystallite diameter of the magnetite irradiated with microwaves calculated from the main peaks of magnetite using the Scherrer equation. From the results, the crystallite diameter of the samples has a minimum value of 33.1 nm at 500 °C, which is significantly lower than that of the raw material and samples irradiated with microwaves at other temperatures. Therefore, the decrease in the crystallite diameter of magnetite occurred most at 500 °C.

**Figure 3:**
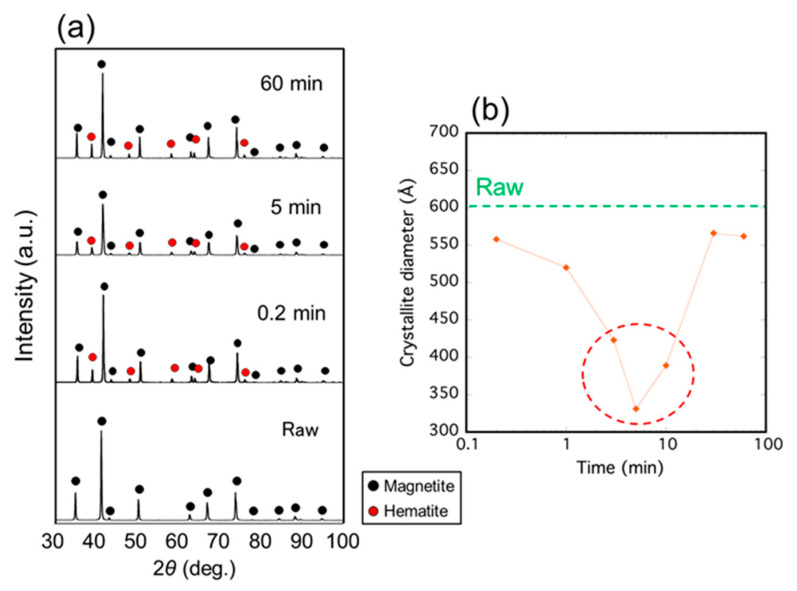
(**a**) XRD patterns of the samples irradiated with microwave at 500 °C for various durations; (**b**) Crystallite diameter of the magnetite calculated by the Scherrer equation as a function of irradiation time.

Figure 3a shows the XRD profiles of the samples irradiated with microwaves at 500 °C for various durations from 0.2 to 60 min. Magnetite was observed in all samples, including the raw material, and the hematite phase was observed in all samples irradiated with microwaves. The crystallite diameter of the microwave-irradiated magnetite, as shown in Figure 3b, indicated that the minimum value was exhibited for a 5 min duration.

**Figure 4:**
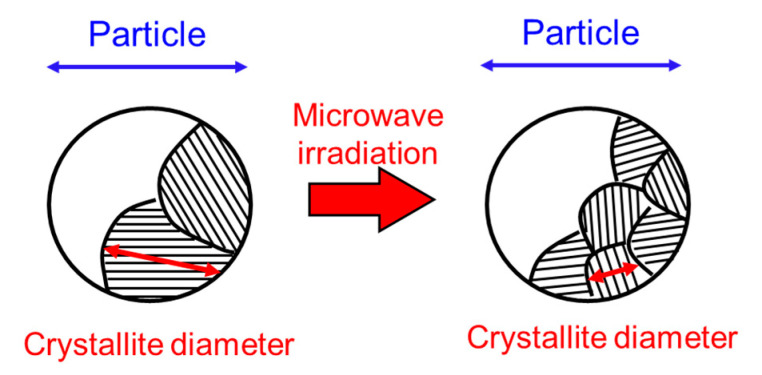
Schematic view of a decrease in the crystallite diameter.

Here, we discuss whether the decrease in the crystallite diameter of magnetite irradiated with microwaves is a non-thermal effect. One can consider that it may be caused by a decrease of the lattice volume accompanied by the reduction of the magnetite [28]. Although the lattice constants of the magnetite irradiated with microwaves were slightly decreased, suggesting the reduction of magnetite, the decrease in the lattice constant of the magnetite is estimated to be no higher than 0.4%. Therefore, the decrease in the crystallite diameters, as shown in Figure 2b and Figure 3b, cannot be explained by a decrease in the lattice constant and caused by a division of the total volume into more crystallites with a consequent increase in the number of grain boundaries as shown in Figure 4. In addition, the temperature during processing is considerably lower than the melting point of magnetite (1597 °C), indicating that it does not occur via the liquid phase. Therefore, it is considered that the decrease in the crystallite diameter of magnetite was caused by a non-thermal effect.

**Figure 5:**
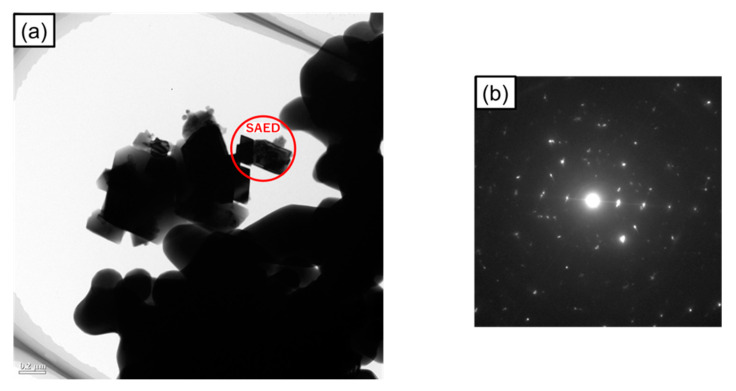
(**a**) TEM image of the sample irradiated with microwaves at 500 °C for 5 min; (**b**) Selected area electron diffraction (SAED) patterns of the magnetite grains.

Figure 5a shows a transmission electron microscopy (TEM) image of the sample irradiated with microwaves at 500 °C for 5 min. From the image, the sample was composed of 0.1–1 mm size grains. Figure 5b shows the selected area electron diffraction (SAED) patterns of the magnetite grains. No ring pattern derived from the 5–20 nm nanodomain structure was observed, as in a previous study [25]. Considering that the XRD patterns from crystalline magnetite were observed, it indicates that a decrease in the crystallite diameter of microwave irradiated magnetite is not de-crystallization but its similar phenomenon, and the crystallite diameter of magnetite in this study is larger than that reported previously. The difference in the crystallite diameters will be discussed in detail later.

### 3.2. Magnetic Properties

**Figure 6:**
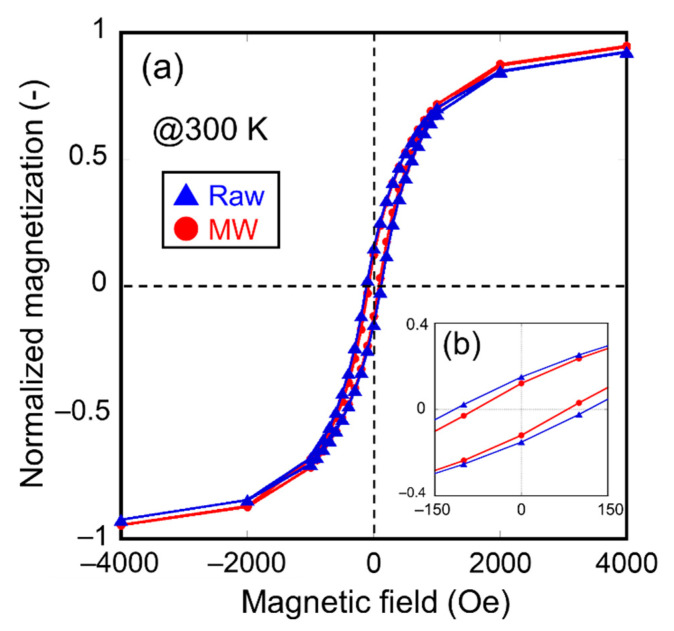
(**a**) *M*-*H* loops measured at 300 K of the raw material and the sample irradiated with microwave at 500 °C for 5 min; (**b**) the enlarged view around the coercivities.

**Figure 7:**
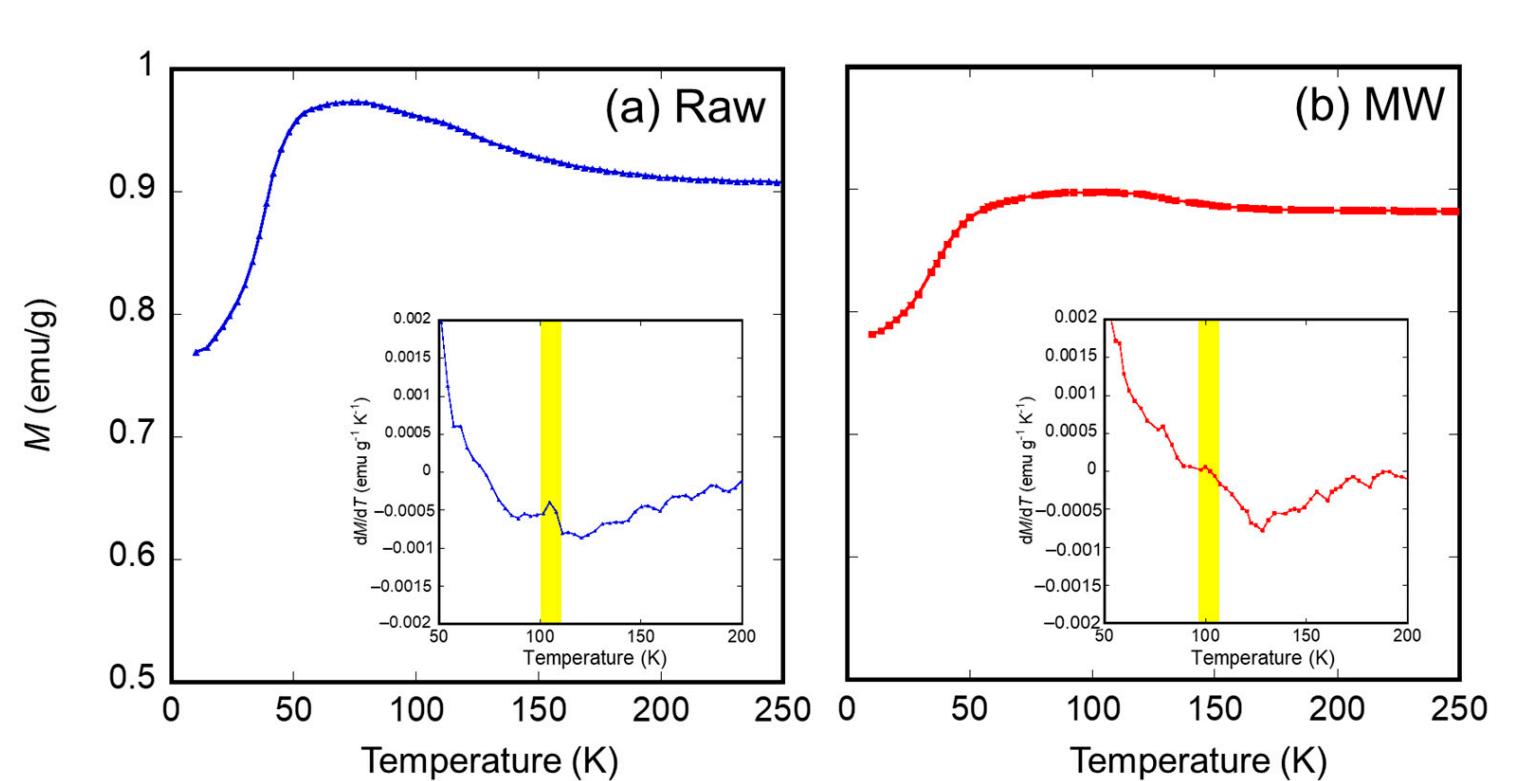
Zero field cooling curve of the (**a**) raw material and (**b**) sample irradiated with microwave (MW) at 500 °C for 5 min. The differentials of the magnetization d*M*/d*T* as a function of temperature are also inserted.

To obtain evidence of the decrease in the crystallite diameter, the magnetic properties of the samples irradiated with microwaves were investigated. First, the authors measured the *M*-*H* loops at room temperature for the raw material and the sample irradiated with microwaves at 500 °C for 5 min, as shown in Figure 6a. An enlarged view of the coercivities is shown in Figure 6b. Here, the magnetization of the samples shown in Figure 6 is normalized with saturation magnetization. The results show that the coercivity of the sample irradiated with microwaves was smaller than that of the raw material. This result indicates that the thermal agitation resistance of the magnetization of microwave-irradiated magnetite is lower than that of the raw material, resulting from a decrease in the crystallite diameter of the sample irradiated with microwaves. Secondary, zero-field cooling (ZFC) curves of the sample irradiated with microwaves at 500 °C for 5 min and the raw material were also measured under 10 Oe applied field, as shown in Figure 7. The differential coefficients of magnetization (d*M*/d*T*) plotted as a function of temperature are shown in Figure 7. Raw material exhibited a characteristic peak of d*M*/d*T* at 105 K. However, a slight peak shift of d*M*/d*T* to 100 K was observed in the case of the microwave-irradiated sample. These peaks can be explained by Verwey transition and metal-insulator transition of the magnetite. Lee et al. reported that the Verwey transition temperature *T*_V_ is dependent on the crystallite diameter of magnetite in the nanosized range and that *T*_V_ decreases when the crystallite diameter of magnetite decreases [29]. Therefore, it is considered that the decrease in the *T*_V_ of the microwave-irradiated sample is due to a decrease in the crystallite diameter, which corresponds to the XRD measurement.

## 4. Discussion

The mechanism of the decrease in the crystallite diameter of magnetite is discussed in this section. First, we considered the temperature dependence of the crystallite diameter. According to Roy et al., de-crystallization of magnetite occurs only at the maximum intensity of the microwave magnetic field in a single-mode cavity [21]. From this, it is considered that the decrease in the crystallite diameter is due to the interaction between the spins of magnetite and the magnetic field of the microwaves. Tanaka et al. simulated the energy loss based on the interaction between the spin of the magnetite and the magnetic field of microwaves and reported that the energy loss takes a maximum value at 500 °C, less than the Curie temperature of magnetite (*T*_C_ = 585 °C) [30]. We hypothesize that the reason why the crystallite diameter of magnetite most decreased at 500 °C was that the energy loss of the interaction between the spin of the magnetite and magnetic field of microwaves was largest at 500 °C. Consequently, microwave energy was efficiently absorbed by magnetite. Most of the absorbed energy is relaxed to thermal energy. However, grain growth will only occur by thermal energy because of the minimization of the boundary energy of the system if sufficient thermal energy is supplied. In a previous study, Fukushima et al. assumed that the grain boundary was formed by *H*-field irradiation to form a nanodomain structure resulting from the de-crystallization phenomena. If this assumption is correct, we require additional energy for forming grain boundaries [31].

We focused on the time dependence of the crystallite diameter to investigate the additional energy required to form grain boundaries. As shown in Figure 3b, the crystallite diameter of the magnetite increased as the irradiation duration increased after 5 min. Focusing on the power profile of the microwave irradiation at 500 °C for 60 min, as shown in Figure 1b, it is observed that the power of the first 5 min was unstable. Then, the power gradually reached a constant value of approximately 600 W. From these results, it is assumed that the absorbed energy from microwaves was used to form grain boundaries, not via thermalization. The decrease in the crystallite diameter occurred only for the first 5 min; nevertheless, microwave energy was used only to maintain the temperature, and the crystallite diameter of magnetite increased over a duration of more than 5 min. Thus, it is suggested that the decrease in the crystallite diameter of magnetite occurred when the energy loss of the interaction between the spin of the magnetite and the magnetic field of microwaves was directly converted into the formation of grain boundaries.

It is sure that the decrease of the crystallite diameter by microwave irradiation was confirmed, a clear difference was acknowledged between the previous study and this study. One of the difference is that the temperature at which the crystallite diameter of the magnetite decreased was considerably lower than that reported in a previous study (>1000 °C), and the crystallite diameter of the magnetite was larger than that of de-crystallized magnetite reported in the previous study [25]. The other is the coercivity of the sample irradiated with microwaves. In the study, the value of the coercivity of the sample irradiated with microwaves was around 100 Oe, but the value was around 1.1 Oe in the previous study [32]. These differences may be caused by a significant difference in the microwave cavity because the TE_103_ single-mode cavity was used for microwave irradiation in the previous study. This difference in the cavities leads to a difference in the quality factor of the cavity, and it is considered that the high-quality factor of the TE_103_ cavity results in a greater decrease in the crystallite diameter of the magnetite than that of the 2.45 GHz microwave cavity used in this experiment. Further study is required to understand the effect of irradiation methods on the microstructure of the materials by comparison with the quality factor of the cavities.

## 5. Conclusions

In this study, the authors investigated the effects of the irradiation temperature and duration of microwave irradiation on the decrease in the crystallite diameter of magnetite. Consequently, in terms of temperature dependence, the crystallite diameter of the magnetite reaches its minimum value at 500 °C, when the energy loss of the interaction between the magnetite spin and the microwave magnetic field becomes maximum. This result indicates that the decrease in the crystallite diameter of magnetite is caused by the interaction between the spin and the magnetic field. In terms of time dependence, it is confirmed that the crystallite diameter of magnetite takes a minimum of 5 min at 500 °C irradiation. Based on this result and the power profile of microwave irradiation, it is suggested that the decrease in the crystallite diameter occurs such that the part of energy loss of the interaction between the spin of magnetite and magnetic field of the microwave is directly converted into the formation of grain boundaries. The TEM image and XRD patterns indicate that magnetite irradiated with microwaves in this study is different from that of the de-crystallized magnetite as shown in the previous study, which may be caused by the difference in the microwave cavity. The sample irradiated with microwaves exhibited lower coercivity, and the Verwey transition shifted to a lower temperature compared to the raw material, which can be evidence of the decrease in the crystallite diameter of magnetite. The new knowledge of the interaction between microwaves and materials suggested in this study will contribute to the further development of the theory of the non-thermal effect. Future work is required to support this consideration; for example, a theoretical approach using dynamical simulation is necessary.

## References

[1] Thostenson E.T., Chou T.-W. (1999). Microwave processing: Fundamentals and applications. Compos. Part A.

[2] Bilecka I., Niederberger M. (2010). Microwave chemistry for inorganic nanomaterials synthesis. Nanoscale.

[3] Menéndez J.A., Arenillas A., Fidalgo B., Fernández Y., Zubizarreta L., Calvo E.G., Bermúdez J.M. (2010). Microwave heating processes involving carbon materials. Fuel Process. Technol..

[4] Beak H., Kashimura K., Fujii T., Tsubaki S., Wada Y., Fujikawa S., Sato T., Uozumi Y., Yamada Y.M.A. (2020). Production of Bio Hydrofined Diesel, Jet Fuel, and Carbon Monoxide from Fatty Acids Using a Silicon Nanowire Array-Supported Rhodium Nanoparticle Catalyst under Microwave Conditions. ACS Catal..

[5] Rybakov K.I., Egorov S.V., Eremeev A.G., Kholoptsev V.V., Plotnikov I.V., Sorokin A.A. (2019). Ultra-rapid microwave sintering employing thermal instability and resonant absorption. J. Mater. Res..

[6] Wojnarowicz J., Chudoba T., Lojkowski W. (2020). A Review of Microwave Synthesis of Zinc Oxide Nanomaterials: Reactants, Process Parameters and Morphologies. Nanomaterials.

[7] Ahmad S., Morsi M.M., Seifert H.J. (2019). Crystallization of two rare-earth aluminosilicate glass-ceramics using conventional and microwave heat-treatments. J. Alloys Compd..

[8] Ano T., Tsubaki S., Liu A., Matsuhisa M., Fujii S., Motokura K., Chun W.-J., Wada Y. (2020). Probing the temperature of supported platinum nanoparticles under microwave irradiation by in situ and operando XAFS. Commun. Chem..

[9] Matsuhisa M., Tsubaki S., Kishimoto F., Fujii S., Hirano I., Horibe M., Suzuki E., Shimizu R., Hitosugi T., Wada Y. (2020). Hole Accumulation at the Grain Boundary Enhances Water Oxidation at α-Fe_2_O_3_ Electrodes under a Microwave Electric Field. J. Phys. Chem. C.

[10] Vaidhyanathan B., Gangi M., Rao K.J. (1996). Microwave-assisted selective deoxygenation of layer- and chain-containing oxides. J. Mater. Chem..

[11] Fukushima J., Takayama S., Goto H., Sato M., Takizawa H. (2017). In situ analysis of reaction kinetics of reduction promotion of NiMn_2_O_4_ under microwave H-field irradiation. Phys. Chem. Chem. Phys..

[12] Chikami H., Fukushima J., Hayashi Y., Takizawa H. (2016). Low-Temperature Synthesis of Aluminum Nitride from Transition Alumina by Microwave Processing. J. Am. Ceram. Soc..

[13] Iwabuchi Y., Fukushima J., Sakuma N., Ito M., Shimo Y., Kishimoto H., Takizawa H. (2016). Oriented texture formation of crystallized Nd_2_Fe_14_B through a microwave heating process. J. Alloys Compd..

[14] Nushiro K., Kikuchi S., Yamada T. (2013). Microwave effect on catalytic enantioselective Claisen rearrangement. Chem. Commun..

[15] Rybakov K.I., Olevsky E.A., Semenov V.E. (2012). The microwave ponderomotive effect on ceramic sintering. Scr. Mater..

[16] Dudley G.B., Richert R., Stiegman A.E. (2015). On the existence of and mechanism for microwave specific reaction rate enhancement. Chem. Sci..

[17] Yanagawa A., Kajiwara A., Nakajima H., Quéméner E.D.-L., Steyer J.-P., Lewis V., Mitani T. (2020). Physical assessments of termites (Termitidae) under 2.45 GHz microwave irradiation. Sci. Rep..

[18] Kimura T., Takizawa H., Uheda K., Endo T. (2000). Microwave Synthesis of X-rays Amorphous Ferrites and the Magnetic Properties. Proc. Int. Conf. Microw. Chem..

[19] Takizawa H., Iwasaki M., Kimura T., Fujiwara A., Haze N., Endo T. (2002). Synthesis of Inorganic Materials by 28 GHz Microwave Irradiation. Trans. Mater. Res. Soc. Jpn..

[20] Takizawa H. (2018). Survey of new materials by solid state synthesis under external fields: High-pressure synthesis and microwave processing of inorganic materials. J. Ceram. Soc. Jpn..

[21] Roy R., Peelamedu R., Hurtt L., Cheng J., Agrawal D. (2002). Definitive experimental evidence for Microwave Effects: Radically new effects of separated E and H fields, such as decrystallization of oxides in seconds. Mater. Res. Innov..

[22] Peelamedu R., Roy R., Agrawal D., Drawl W. (2004). Field decrystallization and structural modifications of highly doped silicon in a 2.45-GHz microwave single-mode cavity. J. Mater. Res..

[23] Nozariasbmarz A., Dsouza K., Vashaee D. (2018). Field induced decrystallization of silicon: Evidence of a microwave non-thermal effect. Appl. Phys. Lett..

[24] Roy R., Fang Y., Cheng J., Agrawal D.K. (2005). Decrystallizing Solid Crystalline Titania, Without Melting, Using Microwave Magnetic Fields. J. Am. Ceram. Soc..

[25] Takayama S., Kakurai K., Takeda M., Matsubara A., Nishihara Y., Nishijo J., Sano S., Nishi N., Sato M. (2009). Investigation of crystal structure formation under microwave heating. Nucl. Instrum. Methods Phys. Res. A.

[26] Yoshikawa N., Cao Z., Louzguin D., Xie G., Taniguchi S. (2009). Micro/nanostructure observation of microwave-heated Fe_3_O_4_. J. Mater. Res..

[27] Takeuchi T., Fukushima J., Hayashi Y., Takizawa H. (2017). Synthesis of Ti_4_O_7_ Nanoparticles by Carbothermal Reduction Using Microwave Rapid Heating. Catalysts.

[28] Yamamoto S., Ruwan G., Tamada Y., Kohara K., Kusano Y., Sasano T., Ohno K., Tsujii Y., Kageyama H., Ono T. (2011). Transformation of Nano-to Mesosized Iron Oxide Cores to α-Fe within Organic Shells Preserved Intact. Chem. Mater..

[29] Lee J., Kwon S.G., Park J.-G., Hyeon T. (2015). Size Dependence of Metal−Insulator Transition in Stoichiometric Fe_3_O_4_ Nanocrystals. Nano Lett..

[30] Tanaka M., Kono H., Maruyama K. (2009). Selective heating mechanism of magnetic metal oxides by a microwave magnetic field. Phys. Rev. B.

[31] Fukushima J., Kashimura K., Takayama S., Sato M. (2012). Microwave-energy Distribution for Reduction and Decrystallization of Titanium Oxides. Chem. Lett..

[32] Takayama S., Fukushima J., Nishijo J., Saito M., Sano S., Sato M. (2012). Sintering of Soft Magnetic Material under Microwave Magnetic Field. Phys. Res. Int..

